# A Transfectable Fusagravirus from a Japanese Strain of *Cryphonectria carpinicola* with Spherical Particles

**DOI:** 10.3390/v14081722

**Published:** 2022-08-04

**Authors:** Subha Das, Sakae Hisano, Ana Eusebio-Cope, Hideki Kondo, Nobuhiro Suzuki

**Affiliations:** Institute of Plant Science and Resources, Okayama University, Okayama 710-0046, Japan

**Keywords:** *Cryphonectria carpinicola*, *Cryphonectria parasitica*, fusagravirus, fungal virus, dsRNA, spherical virion, transfection

## Abstract

A novel dsRNA virus (Cryphonectria carpinicola fusagravirus 1, CcFGV1), isolated from a Japanese strain (JS13) of *Cryphonectria carpinicola*, was thoroughly characterized. The biological comparison of a set of isogenic CcFGV1-infected and -free (JS13VF) strains indicated asymptomatic infection by CcFGV1. The sequence analysis showed that the virus has a two open reading frame (ORF) genome of 9.6 kbp with the RNA-directed RNA polymerase domain encoded by ORF2. The N-terminal sequencing and peptide mass fingerprinting showed an N-terminally processed or degraded product (150 kDa) of the 5′-proximal ORF1-encoded protein (1462 amino acids) to make up the CcFGV1 spherical particles of ~40 nm in diameter. Interestingly, a portion of CcFGV1 dsRNA co-fractionated with a host protein of 70 kDa. The purified CcFGV1 particles were used to transfect protoplasts of JS13VF as well as the standard strain of an experimental model filamentous fungal host *Cryphonectria parasitica*. CcFGV1 was confirmed to be associated with asymptomatic infection of both fungi. RNA silencing was shown to target the virus in *C. parasitica*, resulting in reduced CcFGV1 accumulation by comparing the CcFGV1 content between RNA silencing-competent and -deficient strains. These results indicate the transfectability of spherical particles of a fusagravirus associated with asymptomatic infection.

## 1. Introduction

The family “Fusagraviridae” was proposed in 2016 [1] to accommodate double-stranded (ds) RNA viruses such as Phlebiopsis gigantea large virus 2 (PgV2), Diplodia scrobiculata RNA virus 1 (DsRV1), Fusarium graminearum virus 3 (FgV3), Fusarimu virguliforme virus 1, Sclerotinia sclerotiorum non-segmented virus L (SsNsV-L), and other related viruses [2,3,4,5]. An increasing number of similar virus genomic sequences have been reported largely from filamentous fungi [6,7,8,9], but also from oomycetes (Phytophthora infestans RNA virus 3, PiRV3) [10], plants (papaya meleira virus, PMeV) [11], and insects (Spissistilus festinus virus 1, SpFV1 and Circulifer tenellus virus 1, CiTV1) [12]. Commonly, these viruses have an undivided dsRNA genome with two open reading frames (ORFs), as in the case for many totiviruses (family *Totiviridae*) and “toti-like” dsRNA viruses. The 3′-proximal ORF encodes the RNA-directed RNA polymerase (RdRP) domain and is assumed to be expressed by −1 ribosomal frameshifting, similar to toti- and toti-like viruses [13,14]. However, little or no information is available about the 5′-proximal ORF, while many “toti-like” viruses encode their capsid proteins on the 5-proximal ORFs to form icosahedral particles.

There are few reports on the biological characterization of fungal fusagraviruses, largely because of the lack of methods for their experimental introduction. Therefore, no viral etiology is established for many fusagravirus infections. This contrasts with many other fungal dsRNA viruses such as the members of orders *Durnavirales* (partitiviruses), *Ghabrivirales* (victoriviruses, megabirnaviruses, chrysoviruses, and megatotiviruses), and *Reovirales* (mycoreoviruses), respectively, for which virion transfection has been developed [15,16,17,18,19,20]. Related to this drawback is the ambiguity as to whether fusagraviruses are capsidless or encapsidated dsRNA viruses. Spear et al. suggested the capsidless nature of insect fusagra-like viruses [12], whereas plant fusagraviruses have been shown to encode capsid protein on the 5′-proximal ORF [11]. In contrast to many other fungal dsRNA viruses, a capsidless nature was also suggested for a few fungal fusagraviruses [2,5]. In this sense, we have previously developed a method to examine whether a virus of interest is capsidless, associated with any host proteins, protected by host-derived membrane vesicles, or encapsidated [21,22].

**Figure 1:**
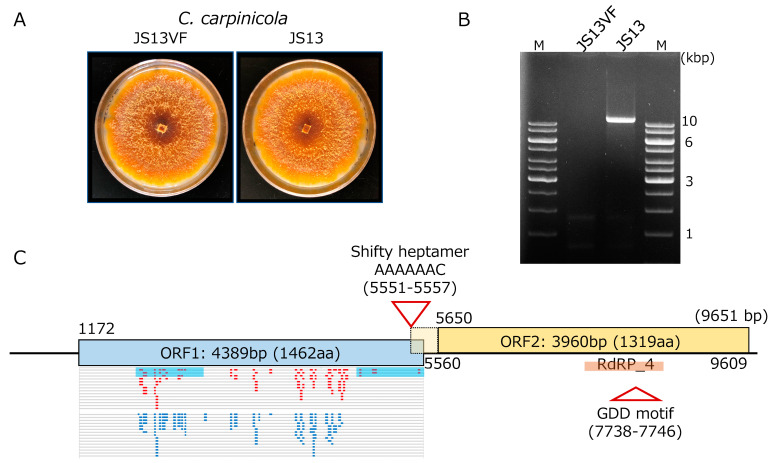
Characterization of CcFGV1 in *C. carpinicola* strain JS13. (**A**) Colony morphology of *C. carpinicola* strain JS13 infected by CcFGV1 and CcFGV1-free isogenic strain JS13VF. JS13VF was obtained via single conidia isolation. The two strains were cultured on PDA for seven days. (**B**) DsRNA pattern of *C. carpinicola* strains JS13 and JS13VF. Purified dsRNA from JS13VF and JS13 were electrophoresed in 1.0% agarose gel in 1× TAE buffer. M in this figure and agarose gels in the following figures (see Figures 3 and 5) refer to DNA ladders used as size standards (Thermo Fisher Scientific., Inc., Waltham, MA, USA). (**C**) Genome organization of CcFGV1. CcFGV1 is 9651 bp in length and possesses two ORFs. Map positions of tentative initiation and stop codons for the ORFs are shown beneath and above the diagram. The relatively long 5′-untranslated region predicted multiple mini-cistrons. The ORF2-encoded protein contains the RdRP_4 domain in the middle. Two truncated ORF1-encoded protein fragments, which were over-expressed in *Escherichia coli* and used as antigen, are denoted by blue filled boxes. Mapping of peptide fragments derived from virus particle fractions (150 kDa and 100 kDa bands, see Figure 4) are shown in small red and light blue bars.

The genus *Cryphonectria* contains an important plant pathogen, *Cryphonectria parasitica,* causing one of the three most destructive tree diseases in addition to other plant pathogenic and non-pathogenic fungi [23,24,25]. It is noteworthy that *C. parasitica* serves as a model filamentous fungal host suitable for studying host antiviral RNA silencing/viral counter-defense responses [26,27]. These *Cryphonectria* spp. are often sympatric to each other. *C. parasitica,* in particular, has been studied extensively from perspectives of biocontrol research, phytopathology, and virology [23,26]. Several research groups have searched large collections of *C. parasitica* isolates from different localities and discovered a variety of viruses [28,29,30,31]. *Cryphonectria* *nitschkei* and *Cryphonectria naterciae*, much less studied than *C. parasitica*, were also found to naturally host several viruses [9,16,32]. Milgroom and colleagues screened a collection of *C. parasitica* and *C. nitschkei* collected from Japan and China and discovered unidentified RNA viruses [28]. Among them, a Japanese strain, JS13, was originally identified as *C. nitschkei*, and the authors claimed it to be the strain infected with an RNA virus unrelated to hypoviruses most prevailing in *C. parasitica* (family *Hypoviridae*, well-known *Cryphonectria* viruses with a positive-sense, single-stranded (+)ssRNA genome) [33]. This fungal strain was re-classified as *Cryphonectria carpinicola* (see Figure 1), which is a recently established species within the genus *Cryphonectria* [25].

In the current study, we report on the thorough characterization of a novel fusagravirus, termed Cryhonectria carpinicola fusgravirus 1 (CcFGV1), from strain JS13 of *C. carpinicola*. Furthermore, we developed a transfection method with spherical particles of CcFGV1, which allowed us to conclude its asymptomatic nature in *C. parasitica* as well as in the original host.

## 2. Materials and Methods

### 2.1. Fungal Strains

*C. carpinicola* strain JS13 (MYA-4105), originally isolated by Milgroom and colleagues from Jisse, Funai District, Kyoto Prefecture, Japan [28], was purchased from American Type Culture Collection (ATCC, Manassas, VA, USA). The standard strain of *C. parasitica* (EP155) and an RNA silencing-deficient mutant Δ*dcl2* [34,35] were generously provided by Dr. Donald L. Nuss (Institute for Bioscience and Biotechnology Research, University of Maryland, Rockville, MD, USA). Strain JS13 infected by Rosellinia necatrix partitivirus 11 (RnPV11, a betapartitivirus) [36] was prepared by protoplast fusion [16] between RnPV11-infected EP155 and a dsRNA-free strain (JS13VF) that was obtained from JS13 via single conidial isolation (see below) with purified virus particles. The absence of a JS13 mycovirus in conidial sub-isolates was confirmed by colony-direct one-step RT-PCR [37]. *Fusarium oxysporum* strain 7n infected by hadaka virus 1 (HadV1, a capsidless RNA virus, family *Hadakaviridae*) was previously described [22]. EP155 infected by the prototypic hypovirus Cryphonectria hypovirus 1-EP713 (CHV1-EP713) was a generous gift from Dr. Donald L. Nuss [38].

Fungal strains were cultured on Difco potato dextrose agar (PDA, Becton, Dickinson & Co., Mountain View, CA, USA) or PDA containing 40 μg/mL hygromycin (PDA-Hyg), unless otherwise mentioned.

### 2.2. Sequence Determination of CcFGV1 and Northern Blotting

CcFGV1 strain isolated from *C. carpinicola* JS13 was partially characterized by Liu et al. [28]. The virus was described a non-hypo virus based on the observation that the CcFGV1 genomic dsRNA could not hybridize with any of CHV1-, CHV2-, CHV3-, or CHV4-specific probes in northern blotting. In this study, dsRNA was purified from strain *C. carpinicola* JS13 and used as a template for cDNA library construction using a non-PCR-based conventional method, as described by Lin et al. [39]. After determining the sequences of cDNA clones (at least three clones with the same region were sequenced), we designed primer sets for 3′-RNA ligase-mediated rapid amplification of cDNA ends (3′-RLM-RACE). The complete nucleotide sequence of the CcFGV1 genome was deposited in the GenBank/DDBJ/ENA database under accession number LC651180. The sequences of oligonucleotides used in RACE are listed in Supplementary Table S1.

Total RNA was purified from PDA-cellophane cultures as described by Eusebio-Cope and Suzuki [40], electrophoresed in agarose gel, and denatured in alkaline conditions. Specific detection of CcFGV1 RNA was carried out according to the method of Sato et al. [22] using a digoxigenin (DIG)-labeled PCR-amplified DNA probe (Table S1).

### 2.3. Sequence and Phylogenetic Analyses

Sequence data were analyzed using GENETX ver. 19 (GENETYX, Tokyo, Japan). Sequence similarity searches were performed with the BLAST program available from NCBI (nucleotide or protein collection) (http://blast.ncbi.nlm.nih.gov/Blast.cgi, accessed on: 10 July 2022). For the phylogenetic analysis, a maximum-likelihood (ML) tree was constructed, as described by Kondo et al. [41,42]. A multiple amino acid sequence alignment based on the RdRP sequences of fusagraviruses was obtained with MAFFT online version 7 (https://mafft.cbrc.jp/alignment/server/, accessed on: 22 June 2022) [43]. The poorly aligned regions were eliminated using trimAl version 1.3 (http://phylemon.bioinfo.cipf.es, accessed on: 22 June 2022) [44] and then used to generate an ML tree using PhyML3.0 (http://www.atgc-montpellier.fr/phyml/, accessed on: 22 June 2022) [45] with the best fit model [46]. Insect-associated fusagra-like viruses, together with a fusagra-like plant virus (persimmon latent virus), were also included in this analysis. Other dsRNA mycoviruses (chrysoviruses, a megabirnavirus, and a phlegivirus) within the order *Ghabrivirales* were used as outgroups. The midpoint rooting tree was refined using FigTree ver. 1.3.1.

### 2.4. Preparation of Polyclonal Antibodies to CcFGV1 ORF1-Encoded Protein and Western Blotting

To prepare antibodies against the CcFGV1 ORF1-encoded protein (150 kDa protein), the N- and C-terminal, 300 amino acids were over-expressed in *Escherichia coli* as glutathione S-transferase (GST) fusion products using an expression vector, pCold (Takara Bio Inc., Otsu, Japan). The native form of both the fusion proteins was purified by GST chromatography according to the manufacturer’s instructions after being highly expressed at 15 °C. A denatured form of both the fusion proteins was purified from preparative SDS-polyacrylamide gel electrophoresis (SDS-PAGE) gel as described earlier. Equal amounts of the N-terminal (denatured) and C-terminal (native and denatured) polypeptides were mixed, and the final concentration was adjusted to 1 mg/mL. Similarly, denatured recombinant protein mixtures were prepared. These preparations were injected into one New Zealand white rabbit (*Oryctolagus cuniculus*) six times at a two-week interval, after being mixed with complete (first injection) and incomplete adjuvants (subsequent five injections).

Western blotting was carried out as described earlier [21]. Proteins were fractionated in 10% SDS-PAGE gel and transferred to a polyvinylidene difluoride (PVDF) membrane (Immobilon-P, Merck Millipore, Billerica, MA, USA). The blot was treated with anti-150 kDa protein antiserum and 1000-fold diluted, mouse anti-rabbit IgG conjugated with horseradish peroxidase, HRP (formerly Kirkegaard & Perry Laboratories, Inc., Gaithersburg, MD. USA). Specific interactions were visualized using ECL Prime Western Blotting Detection Reagent (Cytiva Amersham, Amersham, UK) and a LAS4000 chemical luminescence detection system.

### 2.5. Virion Purification, Protein Analyses, and Transfection

CcFGV1-infected mycelia (approximately 10 g, fresh weight) from JS13 was harvested (from 10-day-old cellophane-PDA culture), ground to a fine powder in the presence of liquid nitrogen, and homogenized in 40 mL of 0.1 M sodium phosphate buffer (PB), pH 7.0, 40 µL of 2-mercapto ethanol, and 1/4 the volume of CCl_4_. This homogenate was centrifuged at 3.5 kilo-rotations per minute (krpm) in a KUBOTA RS-240 rotor (Kubota Co., Tokyo, Japan) for 10 min. The supernatant was centrifuged at 36 krpm in a Beckman 70Ti rotor for 1.5 h. The pellet was resuspended in up to 1 mL of 0.05M PB and used as the crude particle fraction (CPF) in transfection (see below). The CPF was subjected to sucrose density gradient (SGD, 10–50%) centrifugation at 36 krpm for 1.5 h for further particle purification in a Beckman SW41Ti swing rotor. The gradient was fractionated (12 per tube), diluted with about 3 volumes of 0.05 M PB, and ultracentrifuged at 36 krpm for 1.5 h. After being resuspended in 100 µL of 0.05 M PB, each fraction was analyzed for RNA and protein components or subjected to electron microscopy and transfection. Purified particles were negatively stained with an EM stain (Nissin EM Co., Tokyo, Japan) [47] and observed using a model H-7650 transmission electron microscope (Hitachi, Tokyo, Japan).

Peptide mass fingerprinting (MS/MS analysis) was carried out at the Department of Instrumental Analysis and Cryogenics, Advanced Science Research Center, Okayama University (Okayama, Japan). Proteins in CcFGV1 fractions were loaded for 10% SDS-PAGE and the gel was stained with the Rapid Stain CBB Kit (Nacalai Tesque Inc., Kyoto, Japan) or SilverQuest^TM^ Silver Staining Kit (Thermo Fisher Scientific Inc., Waltham, MA, USA), while dsRNA was analyzed by agarose gel electrophoresis in the 0.5× TAE buffer system. Each protein band of interest was subjected to in-gel tryptic digestion according to the manufacturer’s instructions (Bruker Daltonics Inc., Billerica, MA, USA). The digested peptides were subjected to LC-MS/MS analysis with the HPLC-Chip/QTOF system (Agilent Technologies Inc., Santa Clara, CA, USA) at the Department of Instrumental Analysis and Cryogenics, Okayama University, and identified using Mascot software (Matrix Science Inc., Boston, MA, USA).

For N-terminal amino acid sequencing, proteins separated on an SDS-PAGE gel were transferred onto a PVDF membrane, followed by amido black staining. The CcFGV1 150 kDa band (see Section 3) was then excised from the membrane and processed for amino acid sequences on a gas-phase protein sequencer Shimadzu Model PPSQ-31A (Kyoto, Japan) at the Department of Instrumental Analysis and Cryogenics, Okayama University.

Protoplasts of virus-free fungal strains of *C. carpinicola* and *C. parasitica* (wild-type and Δ*dcl2* strains) were prepared as described by Churchill et al. [48]. Three virus sources (10 µL) including CPF, SDG fractions, and purified dsRNA were used to transfect 100 μL of protoplasts (~5 × 10^6^).

### 2.6. RNase A Assay

The RNase assay was performed according to the method of Sato et al. [22] with a slight modification. Fungal mycelia were homogenized in four volumes (*v*/*w*) of 0.05 M PB, after grinding in the presence of liquid N_2_, followed by the removal of mycelial debris using CCl_4_. The homogenate was treated with or without RNase A (10 µg/mL) (Sigma-Aldrich Co. LLC, Milwaukee, WI, USA) for 30 min at 37 °C. RNase A was inactivated with phenol, followed by phenol/chloroform/isoamyl alcohol (25:24:1) and chloroform/isoamyl alcohol (24:1) treatment. DsRNA in the aqueous phase was isolated by cellulose column chromatography, precipitated with iso-propanol, and then subjected to agarose gel electrophoresis.

## 3. Results

### 3.1. Asymptomatic Infection of C. carpinicola by CcFGV1

An isolate (JS13VF) judged as a CcFGV1-free strain by dsRNA gel electrophoresis, and RT-PCR was obtained from the originally CcFGV1-infected JS13 strain of *C. carpinicola* via single spore isolation (Figure 1A). Of 43 single conidial isolates tested for the presence of CcFGV1 dsRNA, only one tested negative for CcFGV1 when examined by dsRNA gel electrophoretic analysis (Figure 1B) and colony-direct one-step RT-PCR (data not shown). As reported by Liu et al. [28], strain JS13 was found to carry dsRNA of approximately 10 kbp. A comparison of the isogenic JS13 and JS13VF suggested that CcFGV1 causes asymptomatic infection in *C. carpinicola.* The two strains showed indistinguishable phenotypes three and seven days post-inoculation onto PDA. The older cultures produced orange pigments (Figure 1A) much more than the younger ones or the standard *C. parasitica* EP155 strain (data not shown).

### 3.2. Molecular Properties of the CcFGV1 Genome

Thirty-six independent cDNA clones, which had over 1.5 kbp inserts, were selected from the library, and subjected to Sanger sequencing. Consequently, a long contig of approximately 9 kbp was obtained after assembly. From the size of the CcFGV1 genomic dsRNA expected from agarose gel electrophoresis (Figure 1B), the contig appeared to span near the full-length genomic sequence. Thus, the remaining terminal sequences were determined by the RACE method, in which at least five independent RACE clones were sequenced. The dsRNA genome of CcFGV1 strain JS13 is 9651 bp in length, with two large ORFs encoding 1462 and 1319 amino acids, respectively. The protein encoded by ORF2 possesses the RdRP domains (Figure 1C). The CcFGV1 genome has a 5′-AAAAAAC-3′ heptamer immediately upstream of the termination codon of ORF1 at map positions 5551–5557. This heptamer is expected to mediate −1 ribosomal frameshifting as predicted for other members of some groups within the order *Ghabrivirales* [49]. Another non-canonical translation is predicted to occur for ORF1, based on the long 5′-untranslated region of 1171 nucleotides with over 10 small ORFs (6~204 nt in size). Internal Ribosomal Entry Site (IRES)-mediated translation is most likely involved in this virus as for other fungal dsRNA and (+)ssRNA viruses [50].

The BLASTp search showed that the ORF1- and ORF2-encoded proteins share the highest amino acid sequence identity (approximately 50%, e-value 0) to those of Cryphonectria naterciae fusagravirus 1 (CnFGV1) (Tables S2 and S3). There were lower levels of amino acid sequences similarity detected between the two CcFGV1 proteins and the counterparts of other previously reported fusagraviruses such as Fusarium virguliforme dsRNA mycovirus 1 (FvMV1) and Trichoderma atroviride mycovirus 1 (TaMV1) (Tables S2 and S3).

### 3.3. Phylogenetic Analysis of Fusagraviruses

**Figure 2:**
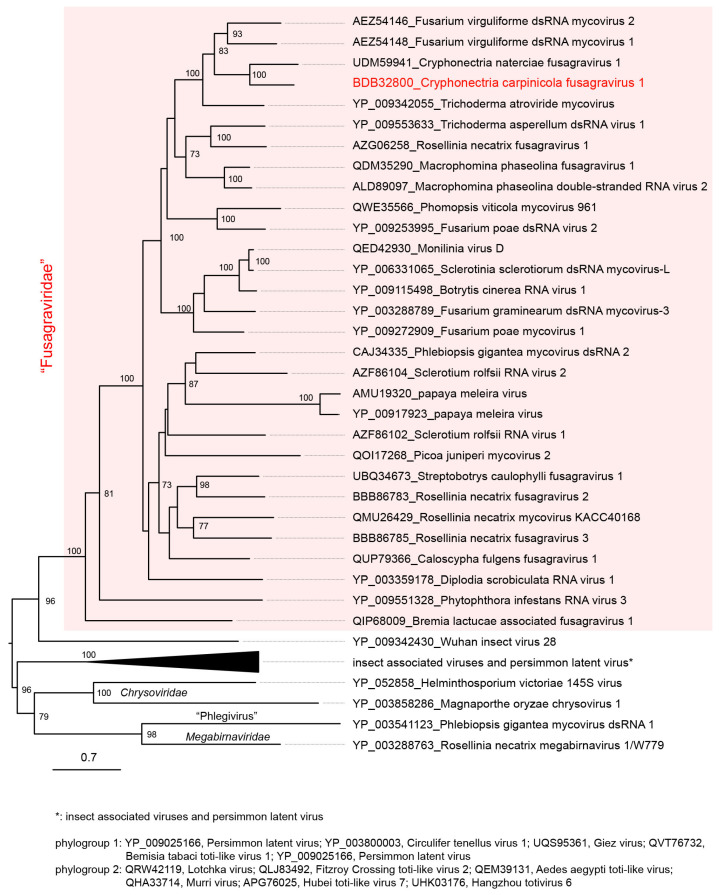
Phylogenetic analysis of CcFGV1. A maximum likelihood (ML) tree was generated based on the alignment of RdRP sequences using a MAFFT alignment. The model Q.pfam +R+F was selected as a best-fit model for the alignment. The virus names are followed by the GenBank accession or Ref-seq numbers of their sequences. The insect-associated viruses and persimmon latent virus (a plant virus) related to fusagraviruses are displayed in a collapsed state. Other dsRNA mycoviruses (chrysoviruses, a megabirnavirus, and a phlegivirus) were used as outgroups. The midpoint rooting tree was refined using FigTree ver. 1.3.1. Numbers at the nodes indicate bootstrap values of >70%.

To understand the phylogenetic relationships between CcFGV1 and other fusagraviruses, an ML tree based on RdRP (ORF2 protein) sequences was constructed (Figure 2). The analysis confirmed that CcFGV1 and other fusagraviruses formed a clade with a strong statistical support value (100%) and were distantly related to a group of insect-associated viruses and a plant virus infecting persimmon. CcFGV1 sub-clustered with fusagraviruses from *C. naterciae* (CnFGV1), *F. virguliforme* (FvMV1 and FvMV2), and *T. atroviride* (TaMV1). This relationship was congruent with the above-mentioned BLASTp result using CcFGV1 ORF2 protein as the query. A similar topology was also observed in the ML tree based on the ORF1-encoded proteins (data not shown).

### 3.4. CcFGV1 dsRNA Is Inaccessible by RNase in Mycelial Homogenate

**Figure 3:**
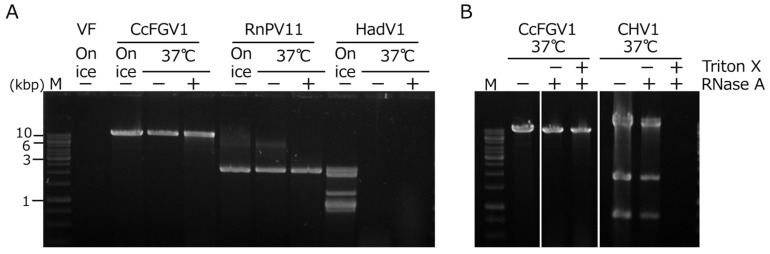
RNase A treatment of mycelial extracts containing viral RNAs. (**A**) *C. carpinicola* strain JS13 infected CcFGV1, *C. carpinicola* strain JS13VF infected by RnPV11 (encapsidated virion, a partitivirus), and *F. oxysporum* strain 7n infected by HadV1 (capsidless dsRNA replicative form, a hadakavirus) were used. Mycelial homogenates were incubated at 37 °C or on ice for 30 min in the presence or absence of RNase A. DsRNA fractions were isolated as described in Section 2, and subsequently electrophoresed on a 1.0% agarose gel. (**B**) Effects of treatment by the non-ionic detergent Triton X-100 on RNase susceptibility. Mycelial homogenates were treated as for (**A**) in the presence or absence of Triton X-100.

Whether CcFGV1 formed rigid particles was an interesting question, given that several previously isolated fusagraviruses were reported to be difficult to purify as particles. In order to examine the possibility of the capsidless nature of CcFGV1, we performed an RNase assay developed by Sato et al. [22] for predicting the virus form in mycelial homogenates. We included two fungal RNA viruses: an encapsidated dsRNA virus (a betapartitivirus, RnPV11) and a capsidless (+)ssRNA virus (a hadakavirus, HadV1). The HadV1 dsRNA replicative form was fully digested at 37 °C within 30 min regardless of the addition of RNase A, suggesting that the HadV1 dsRNA replicative form is susceptible to RNase intrinsically present in the host fungus (Figure 3A). RnPV11 genomic dsRNA was encapsidated and was resistant to RNase in mycelial homogenates. CcFGV1 dsRNA behaved in a way similar to RnPV11 genomic dsRNA (Figure 3A), and was found to be present in a form resistant to RNase A.

To examine whether CcFGV1 was enclosed in cytoplasmic lipid vesicles like hypoviruses [51,52], we treated the homogenates with the non-ionic detergent Triton X-100. Consequently, CHV1 dsRNA replicative form became susceptible to RNase A only after treatment with Triton X-100, whereas CcFGV1 genomic dsRNA remained resistant even after being treated with Triton X-100, as in the case for the genomic dsRNA of RnPV11 (Figure 3B and data not shown).

These results suggest that CcFGV1 is encapsidated like other classical dsRNA viruses or tightly associated with proteins like polymycoviruses (multi-segmented dsRNA viruses distantly related to some animal (+)ssRNA viruses such as caliciviruses) [21,53]. This possibility was tested in the following subdivision. This is worthy of note that both encapsidated dsRNA viruses and polymycoviruses can be pelleted by ultracentrifugation, and these pelleted fractions are infectious without any further purification [53].

### 3.5. Particle Fractionation and Identification of Capsid Protein-Coding Region

**Figure 4:**
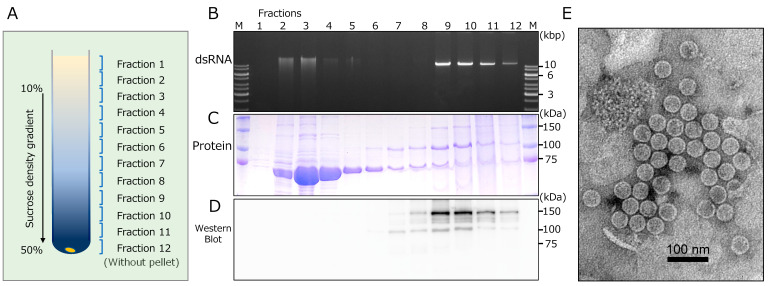
Fractionation and morphology of CcFGV1 particles. Crude CcFGV1 particles were obtained by one round of differential centrifugation and fractionated by sucrose density gradient (SDG, 10–50%) centrifugation. In total, (**A**) 12 separated fractions were examined for dsRNA by (**B**) agarose gel electrophoresis, and protein components by (**C**) SDS-PAGE and (**D**) Western blotting. Agarose gel electrophoresis and SDS-PAGE were carried out using conventional methods. Proteins were stained with Coomassie Brilliant Blue R250. M refers to the protein size markers (Precision Plus Protein Dual Color Standards, Bio-Rad Laboratories, Inc., Hercules, CA, USA). Rabbit polyclonal antibodies against the two fragments of CcFGV1 ORF1-encoded protein were utilized in western blotting. (**E**) Electron micrograph of CcFGV1 in the sample derived from fraction no 9. CcFGV1 particles was observed using a Hitachi H-7100 electron microscope after staining with the EM Stainer (Nissin EM Co., Tokyo, Japan). The scale bar represents 100 nm.

We attempted to purify CcFGV1 particles from the virus-infected strain JS13 of *C. carpinicola* by conventional differential centrifugation and SDG centrifugation. Fractions were monitored for the CcFGV1 genomic dsRNA and protein components after ultracentrifugation. These fractions were subjected to dsRNA and protein analyses, transmission electron microscopy, and transfection of *C. parasitica*. Interestingly, agarose gel electrophoresis showed that dsRNA-rich fractions (fractions 2–4) were observed in a relatively upper zone of the SDG, while the presence of dsRNA was also detected in the fractions from the middle to the bottom portion (fractions 9–12) (Figure 4A,B). Note that a major peak was observed only in the middle portion of the fraction for the encapsidated partitivirus, RnPV11 (data not shown). SDS-PAGE analysis showed that the upper dsRNA-rich zones (fractions 2–4) also contained a major protein band of 70 kDa, while proteins of 100 kDa and 150 kDa were observed in the middle to the bottom zones, including minor dsRNA-containing fractions (fractions 8–11) (Figure 4C). In order to investigate whether the detected proteins were virally encoded, we performed western blotting with an antiserum against two recombinant fragments of ORF1-encoded protein (Figure 1C). As a result, only the 100 kDa and 150 kDa proteins were detected in fractions from the middle to the bottom portion, but not the 70 kDa protein in the upper top fractions (Figure 4C,D).

Electron microscopy revealed spherical particles of approximately 40 nm in diameter in fraction 9 (Figure 4E). However, no such particles were observed in fraction no. 3, which corresponded to the upper dsRNA peak (data not shown). Interestingly, two CcFGV1 dsRNA-containing zones had different SDS-PAGE profiles, but virus-like particles were only detected in the lower dsRNA zone (Figure 4B).

We used the same buffer in RNase A treatment as that for virus particle purification. Therefore, the RNase-resistant nature of CcFGV1 genomic dsRNA (Figure 3) can be partially explained by the result of virion purification. Some portion of CcFGV1 dsRNA in the partially purified preparations was a non-virion form, suggesting that the virus may exist in an RNase A-resistant form possibly associated with the 70 kDa protein, together with a classical virion.

Western blotting revealed that the 100 kDa and 150 kDa proteins were CcFGV1 ORF1-encoded proteins. We carried out peptide fingerprinting and a chemical determination of the terminal amino acid sequences. MS/MS analysis of the 100 and 150 kDa proteins showed that peptide fragments derived from these proteins were mapped to the C-terminal at two-thirds of the ORF1-encoded protein (Figure 1C). Peptide mapping patterns for the 100 and 150 kDa proteins (Figure S1) suggested that the 100 kDa protein was a C-terminally truncated version of the 150 kDa protein. We could determine the N terminal amino acid sequence of the 150 kDa protein to be QFSPSPE---, which was mapped to map positions 264 to 270 of the ORF1-encoded protein.

Taken together, these results suggest that CcFGV1 form spherical particles, the capsid protein of which is encoded by ORF1. Whether the 100 kDa protein is derived by programmed processing or degradation at their C terminus is unclear. This capsid protein-coding profile of CcFGV1 is similar to yado-nushi virus 1 (a toti-like dsRNA virus, the proposed family “Yadonushiviridae”), the capsid protein N-terminus of which starts at around one-third of the ORF1-encoded protein [54].

### 3.6. Purified CcFGV1 Particles Are Trasnfectable and Induce Asymptomatic Infection in Other Cryphonectria Species

Virus transfection was performed on a dsRNA-cured *C. carpinicola* strain JS13VF, and a strain of the different *Cryphonectria* species, *C. parasitica* EP155, and its RNA silencing-deficient mutant Δ*dcl2*. For virus sources, crude particle preparations, deproteinized purified CcFGV1 dsRNA, and SDG fractions (Figure 4) were used. The results are summarized in Table 1. Crude extracts inefficiently provided transfectants, while purified CcFGV1 dsRNA failed to show transfectability. Comparison of the fractions 3 and 4 (dsRNA-rich zone, but no virions) as well as 9 (minor dsRNA peak with virions) obtained by SDG centrifugation (Figure 4) showed that, whereas no transfectants were obtained with fractions 3 and 4, fraction 9 provided a high frequency of transfection of *C. carpinicola*. Particle fractions prepared by cesium chloride equilibrium centrifugation also gave transfectants, albeit inefficiently, compared to SDG fraction 9 (data not shown).

**Figure 5:**
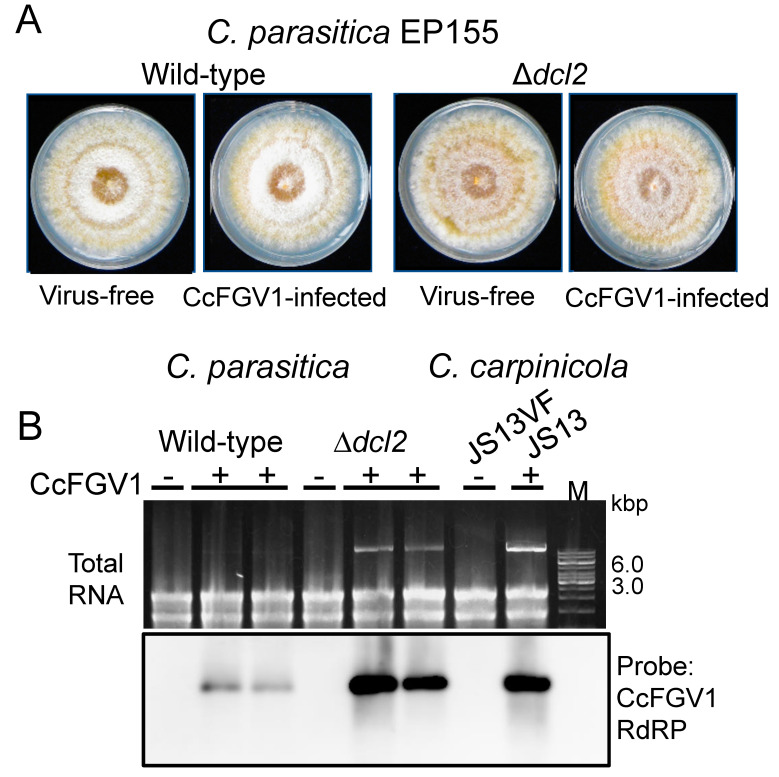
Transfection of *C. parasitica* and CcFGV1 accumulation in the new fungal host strains. *C. parasitica* wild-type strain (EP155) and an RNA silencing-deficient strain Δ*dcl2* were transfected by CcFGV1 crude or purified preparations, or purified dsRNA fractions (Table 1). Two sets of isogenic CcFGV1-free and CcFGV1-infected strains were grown on PDA on the benchtop for five days and photographed (**A**). (**B**) Viral accumulation was compared between three fungal strains (*C. parasitica* EP155 and Δ*dcl2*, and *C. carpinicola* JS13) by agarose gel electrophoresis (top panel) and northern blotting (bottom panel).

Transfection allowed for phenotypic comparison between isogenic CcFGV1-free and CcFGV1-infected fungal strains. Figure 5A shows two sets of such fungal strains. As in the case for *C. carpinicola*, no phenotypic alterations by CcFGV1 were observed in *C. parasitica* standard strain EP155 and the RNA silencing-deficient mutant Δ*dcl2*.

We also compared the virus content in three different fungal strains. In *C. parasitica* EP155, a much lower level of CcFGV1 dsRNA (almost invisible in the total RNA on the agarose gel) was compared to that in *C. parasitica* Δ*dcl2* or *C. carpinicola* JS13 (Figure 5B and data not shown). If the latter two were compared, JS13 provided a slightly thicker dsRNA band. Northern blotting with total RNA fractions confirmed a CcFGV1 RNA accumulation pattern similar to the CcFGV1 dsRNA accumulation pattern shown by the agarose gel electrophoresis (Figure 5B).

## 4. Discussion

The first near-complete or complete fusagravirus genome sequences were reported for PgLV2 and FgV3 from filamentous fungi, *Phlebiopsis gigantea* and *Fusarium graminearum,* in 2009, respectively [2,4]. The number of fusagraviruses genome sequences has grown since then, and this led Wang et al. [1] to propose a new family “Fusagraviridae,” which includes not only fungal viruses, but also other related viruses from plants and oomycetes (Figure 2). However, several virological key questions have been unanswered, such as whether they are encapsidated or what their impact on the host organisms? This study reveals tentative taxonomical placement of, and provides a thorough characterization of a fusagravirus, CcFGV1, and important insights into its virological properties. The closest relative of CcFGV1 is a fusagravirus from *C. naterciae*, associated with a canker disease of cork oak and chestnut in Europe [24]. The family “Fusagraviridae” has not yet officially been approved, and neither family nor species demarcation criteria have been established by the International Committee on Taxonomy of Viruses (ICTV). However, we propose that these viruses belong to two different species based on the RdRP amino acid sequence identity (45.8%, BLASTp) (Table S3) and phylogenetic relationship (Figure 2).

Spherical particles have been purified only for a plant fusagravirus, papaya meleira virus, whose coat protein is encoded by ORF1 [11]. A fungal fusagravirus (TaMV1) was also shown to form isometrical particles of 40 nm in diameter [55]. However, difficulties with particle isolation were noted earlier for fungal fusagraviruses, as well as oomycetous and insect fusagra-like viruses such as PgV2, PiRV3, and SpFV1 [2,10,12]. A capsidless nature is not so unusual for fungal viruses, particularly for (+)ssRNA fungal viruses exemplified by hypoviruses, endornaviruses, narnaviruses, hadakaviruses, and some flexiviruses, although a capsidless nature has not been well-established for dsRNA viruses except for polymycoviruses. It should be emphasized that polymycoviruses form either non-conventional virions (dsRNA-protein complex) or filamentous particles [56,57]. The polymycovirus genomic dsRNA is tightly associated with a virally encoded proline, alanine, and serine-rich protein (PASrp). This form of dsRNA/PASrp is infectious when introduced into host protoplasts [53]. A high content of these three amino acid residues (proline, alanine, and serine) was also noted for the ORF1-encoded protein of PgV2, as observed for capsidless polymycoviruses [53]. These considerations prompted us to suspect a capsidless nature of CcFGV1 at the beginning of this study.

Our RNase assay showed that CcFGV1 dsRNA is in a nuclease inaccessible form in the mycelial homogenates even after treatment with Triton X-100 (Figure 3B). This suggests two possibilities that (1) CcFGV1 dsRNA is encapsidated; or (2) CcFGV1 dsRNA is tightly associated with protein, as in the case for polymycoviruses. Interestingly, there were two peaks for CcFGV1 dsRNA after SDG centrifugation: one near the top of the gradient and the other peak the two-thirds down. The fraction corresponding to the minor dsRNA peak contained infectious spherical particles of ~40 nm in diameter (Figure 4E) made up of mature ORF1-encoded protein (Figure 4C,D). A portion of CcFGV1 dsRNA appeared not to be packaged into spherical particles, and was likely associated with the 70 kDa protein (Figure 4C,D). It is noteworthy that the 70 kDa protein shares sequence identities with cellular alcohol oxidase (S. Hisano and N. Suzuki, unpublished data). Its *Helminthosporium victoriae* ortholog (Hv-p68) was shown earlier to copurify with the genomic dsRNA of a chrysovirus, Helminthosporium victoriae 145S virus, and possesses RNA-binding activities [58]. If the major fraction of dsRNA was derived from the disruption of particles during purification, the dsRNA would be RNase A susceptible. This unusual fractionation pattern of CcFGV1 may explain the difficulty in purifying fusagravirus particles. Generally, phosphate buffer of an approximately neutral pH has frequently been used for the particle extraction of fungal viruses from filamentous fungi [39,59,60]. However, we found that borate buffer at pH 8.0 provided a much better yield of CcFGV1 (S. Hisano and N. Suzuki, unpublished data). Although we cannot rule out the possibility of the capsidless nature of other fusagraviruses reported to be difficult to purify, it is worth trying different purification methods.

The experimental introduction of fungal viruses is necessary for establishing a viral etiology or determining virus effects on host fungal strains. However, this had never been possible for fusagra- or fusagura-like viruses, and no biological outcome of these dsRNA virus infections has been reported. The only exception was a fusagravirus (CnFGV1) from *C. naterciae,* which was shown to cause growth defects in strains of a different *Cryphonectria* species (*C. carpinicola)* through cross-species horizontal transfer via co-culturing [9]. In this study, CcFGV1 from *C. carpinicola* was introduced by virion transfection into the strains of two different *Cryphonectria* species, *C. carpinicola* and *C. parasitica*. Virion transfection has become applicable for diverse dsRNA viruses, including chrysoviruses [16], victoriviruses [18,19], yadonushiviruses [54], botybirnaviruses [61] and megabirnaviruses [14] (*Ghabrivirales*), and partitiviruses (*Durnavirales*) [15,62], since the first successful attempt with a mycoreovirus (*Reovirales*) [63]. This study added another family member that can be used in transfection for horizontal transfer. This transfection method might allow for introduction into any fungal host as long as their protoplasts are available. Unlike CnFGV1, CcFGV1 showed asymptomatic infection in the wild-type strains of the two *Cryphonectria* species (Figure 1 and Figure 5), despite the fact that Δ*dcl2* of *C. parasitica* is deficient in antiviral RNA silencing and is often vulnerable to virus infections [35,64].

Another interesting finding was that even the antiviral RNA silencing-defective *C. parasitica* strain with the EP155 background (Δ*dcl2*) showed no symptoms upon infection by CcFGV1. Generally, even when RNA silencing-competent *C. parasitica* EP155 shows little or mild symptoms upon infection of a virus, pronounced symptoms are induced in Δ*dcl2* by the same virus, as exemplified by (+)ssRNA and dsRNA viruses (e.g., [14,18,36,65]). Thus, the observation shown in Figure 5 is unusual. Thus far, only a few examples have been reported in which symptomless infection was observed in *C. parasitica* Δ*dcl2* despite the fact that a virus of interest is targeted by RNA silencing and its replication is enhanced in Δ*dcl2* as for CcFGV1. Examples include a hypovirus (CHV4) [20] and two *Rosellinia necatrix* partitiviruses (RnPV3 and RnPV18) [36]. Moreover, there is no phenotypic difference between EP155 and Δ*dcl2* infected by Cryphonectria parasitica mitovirus 1 (CpMV1, a mitochondrially replicating virus) [66]. Although CpMV1-derived small RNAs are detected in CpMV1-infected EP155, there is no significant difference in CpMV1 content between EP155 and Δ*dcl2* [66]. Thus, it is unclear whether RNA silencing works to inhibit CpMV1 replication.

Fusagraviruses are often co-detected with other RNA viruses in single host fungi [5,7,32,67,68] in which the possible impacts of fusagraviruses on host fungi and coinfecting viruses are unexplored. This study provides a platform for studying virus–host and virus–virus interactions involving fusagraviruses.

## Figures and Tables

**Table 1:** Transfection efficiency of different virus sources.

Host		Virus Source for Transfection								
Species	Strain	Crude Virus Particles Fraction			Purified dsRNA		Sucrose Density Gradient Fraction ***			
							3		4	9
*C. parasitica*	EP155	2/6 *	1/12 **							
	EP155Δ*dcl2*	2/6 *								
*C. carpinicola*	JS13VF			1/36 *	0/36	0/12	0/36	0/12	0/12	11/12

*, ** Crude virus particle fractions were obtained from mycelial homogenates by centrifugation at 11 krpm for 20 min after stirring at 4 °C in 8% polyethylene glycol 6000 and 1% NaCl for 2 (*) or by one round of differential centrifugation (**) (see Section 2). *** See Figure 4.

## Data Availability

Not applicable.

## Supplementary Materials

The following supporting information can be downloaded at: https://www.mdpi.com/article/10.3390/v14081722/s1, Figure S1: Mascot search results of CcFGV1 structural proteins p150 and p100; Table S1: List of primers used in this study; Table S2: BLASTp search with the CcFGV1 ORF1-encoded protein; Table S3: BLASTp search with CcFGV1 ORF2-encoded RdRP.
