# Supplementary material for: A Transfectable Fusagravirus from a Japanese Strain of Cryphonectria carpinicola with Spherical Particles
Source: Viruses. 2022 Aug 4;14(8):1722. doi: 10.3390/v14081722 (PMC9413294; doi:10.3390/v14081722)
Supplement: Supplementary file 1 [file viruses-14-01722-s001.zip › viruses-1836546-supplementary.pdf]

SUPPLEMENTARY DATA FOR

A transfectable fusagravirus from a Japanese strain of *Cryphonectria carpinicola* with spherical particles

Subha Das[1, 2, †], Sakae Hisano[1, †], Ana Eusebio-Cope[1, 3], Hideki Kondo[1], and Nobuhiro Suzuki[1, *]

[1]Institute of Plant Science and Resources, Okayama University, Kurashiki, Okayama 710-0046[1)], Japan

Short title: A novel transfectable fusagravirus from *Cryphonectria carpinicola*

†These authors contributed equally to this work.

* Correspondence may be sent to N. Suzuki
Institute of Plant Science and Resources, Okayama University
Chuou 2-20-1, Kurashiki, JAPAN
Telephone: 81-86-434-1230
FAX: 81-86-434-1232
E-mail: nsuzuki@okayama-u.ac.jp

Supplementary tables: 3
Supplementary figure: 1

Supplementary Figure S1. Mascot search results of CcFGV1 structural proteins of 150 kDa and 100 kDa.

Match to CcFGV1 ORF1: Score: 927
Found in search of 200519_JS13-150kDa-long.mgf
Calculated pI value: 6.82
NCBI BLAST search of JS13ORF1 against nr
Unformatted sequence string for pasting into other applications
Fixed modifications: Carbamidomethyl (C)
Cleavage by Trypsin: cuts C-term side of KR unless next residue is P Sequence Coverage: 22%

Match to CcFGV1 ORF1: Score: 1634
Found in search of 200519_JS13-100kDa-long.mgf
Nominal mass (M_r): ; Calculated pI value: 6.82
NCBI BLAST search of JS13ORF1 against nr
Unformatted sequence string for pasting into other applications
Fixed modifications: Carbamidomethyl (C)
Cleavage by Trypsin: cuts C-term side of KR unless next residue is P Sequence Coverage: 22%

MASSSSSRSARTLNEQISSLPTRPILQGEVVPSQPNGLCYLNFFHPASHSLFDKTRVWEKP
AEIVQHAISVGAVLVADQKFSVQLGGQYLRKLSSSGPSIEYAHVARSGSWTPAQILSAECF
AETRFGGESHSTGLAPMILSLLLSFFLFIGTAVGGYLWAWQPAHYSSPPNYGPGSCYLVYF
HPLVRPFAFALLGLRPRLWSVRLLALVLPTVSETNLMVQKAFTLADGTAVYHVTACTGGSW
AGFGPRMSLASLDPSARLG**QFSPSPE**DQVIETK**DAPVAKAVTGVPSLQSLGGVRQVPTSLP**
VPQISPGFQRRWSPSTFPASLTSENLVWEHSEDGHLAVK**RVSEFVRHFPEDSAISR**LPQR**F**
AETLLVKWFAR**RDCNEASTVPASYRRSFDRDIVQR**TVLDEFSSIGQVLDNNLTLDVLK**DAD**
SSAAYSRGLGSRNQR**LVPGWEAAVMTR**WLR**FRDFATR**SASAR**SYKEFVFR**LASRFTLATAA
QDIVNSVPDYATHVADTATDVQIIHLNADPIIPAPVAGQPPPPPVWGEAQLWDPAMIQALV
DGR**AQLIDAEGFTK**EEIAQIIGCLAPSGAGNVPILRRSVVPDPATPDATEDVDMLPACARF
TYPNGVTHIIVHHGNSAIPDQADQQWIAAHAFDFPSLAILATVMRTYATR**HALEDLFK**WAF
EAVAYR**TAFYTAADALGAR**HDLHSDLLISAQGASELHLPRNVTGSAYFDTFFAPVQVTGDL
EMYLYATPQQLVTSATLASHCR**AVALAWACKSGSLLGLSFTR**AAAR**QNQFTR**NQQDKWLRM
YYGELNIFSALHANAMGFQYGFAPSALVRR**TEGGLLPDWWK**NYVAPTLVNHYLELWAMQSI
PTFQVLPYYDATAKTSHVQWPEDTPDSTAALPSFNTERKVRLAREFSPLTGHSWLGDGGAE
YNAQFYYAQGADGR**FAYEGAEPKANFSSWTGTFARSFPSTPAAARGISLTNGR**LGEAFSDF
ILPGSFISYRVASDK**VINWGVNQIDDRALNNSEVRRWWLASK**GAAHTSLMVNYVSPISQHY
ELDDFADYSVTLWEKEGR**FAALSFGPLPRLLAPDTFDPVNITRDQSAFNLRFDSKPYALDQ**
LSTSRITHNAR**SDVPASGPQTKYTPAEVNARVAAAISSR**R**NRPSGQVSYQAK**NPIFADQLP
HLNDYSVDVREEGLDFSRPNENSGALPAQDETSSDRLR**QIQEAEAR**LDNAFQEYMADQQQK
RAIRQARVAHVQPTFTPVPIHAPIPQRRR**RLPTVTDNGPPVPVSVPVSR**HKGHMTYSANRP
LQQQAPPVVPNDAAAASVQQMSVNLAELQRRRASLTPTLAGPNGRRAASPRARYLSKRNEA
GQPTARFGDVVHMHHIKEQDAPPTVSSPKPGEPLPDLSWAQPQPFLTNQQPPDPAQALPPQ
PTEEEYPRLANRARPSSAPEQVDFSQIDWDHGSGDAR**AQMLEEFSR**KAHQHVDMTKPKN

Blue: Fragments from the 100kDa protein
Red: Fragments from the 150kDa protein
Purple: Fragments from both 100 and 150kDa protein
Underlined: N-terminally sequenced residues of the 150kDa protein

Supplementary Table S1. List of primers used in this study.

Primer Name	Sequence	CcFGV1 map position	Used in
JS13-V-ctg1-5R	TGGATCCTGGGTCATGAGTGATGA	5927 < 5950	RT-PCR
JS13-V-ctg1-3F	TTGGACACTTACGTGCGATCTCTC	9294 > 9317	RT-PCR
JS13-V-ctg2-5R	CAAGCAAGTGCAACTGCGCG	3432 < 3451	RT-PCR
JS13-V-ctg2-3F	CACATCAAGGAACAGGACGCTCC	5241 > 5263	RT-PCR
JS13-V-ctg3-5R	CCGGGTGTTCCACACCAGGGCCATAACT	259 < 286	RT-PCR
JS13-V-ctg3-3F	TCTACGTTCCCCGCTTCTCTCACATCTG	2129 > 2156	RT-PCR
JS13-V-RACE-5R	CACACAGGACAGATCTTGCCT	579 < 599	RACE
JS13-V-RACE-3F	CATAGCTATAATGCTTCAACGGCCC	9125 > 9149	RACE
3RACE-adaptor	PO4-CAATACCTTCTGACCATGCAGTGACAGTCAGCATG		RACE
3RACE-1st	CATGCTGACTGTCACTGCAT		RACE
3RACE-2nd	TGCATGGTCAGAAGGTATTG		RACE
JS13-V-Plobe-5F	GAGGCCGCGAGAGATCTGAAA	7502 > 7522	DIG-probe preparation
JS13-V-Plobe-3R	CGGCAAGGGGTTGGAGAAGAA	7961 < 7981	DIG-probe preparation

Supplementary Table S2. BLASTp search with the CcFGV1 ORF1-encoded protein.

Description (virus [isolate])	Description (protein)	Max score	Total score	Query cover	E value	Identity	Accession
[Cryphonectria naterciae fusagravirus 1]	putative structural/gag protein	1329	1329	88%	0	54.31	UDM59940.1
[Fusarium virguliforme dsRNA mycovirus 2]	putative structural/gag protein	904	904	71%	0	46.75	AEZ54145.1
[Fusarium virguliforme dsRNA mycovirus 1]	putative structural/gag protein	893	893	72%	0	45.57	AEZ54147.1
[Trichoderma atroviride mycovirus]	putative structural/gag protein	753	753	73%	0	39.46	YP_009342054.1
[Macrophomina phaseolina fusagravirus 2]	hypothetical protein	440	440	71%	3.00E-127	30.84	QKO02083.1
[Macrophomina phaseolina fusagravirus 1]	hypothetical protein	431	431	63%	3.00E-123	32.29	QDM35289.1
[Macrophomina phaseolina fusagravirus 4]	hypothetical protein	423	423	64%	3.00E-121	32.74	QKO02087.1
[Macrophomina phaseolina fusagravirus 5]	hypothetical protein	421	421	64%	1.00E-120	32.40	QKO02089.1
[Trichoderma asperellum dsRNA virus 1]	hypothetical protein	421	421	82%	4.00E-119	27.93	YP_009553632.1
[Macrophomina phaseolina double-stranded RNA virus 2]	hypothetical protein	416	416	64%	5.00E-119	32.12	ALD89096.1
[Fusarium poae dsRNA virus 2]	hypothetical protein	414	414	64%	3.00E-117	30.13	YP_009253994.1
[Macrophomina phaseolina fusagravirus 3]	hypothetical protein	402	402	64%	7.00E-114	31.77	QKO02085.1
[Botrytis cinerea RNA virus 1]	hypothetical protein	397	397	66%	3.00E-112	31.74	YP_009115497.1
[Sclerotinia sclerotiorum fusagravirus 1]	hypothetical protein	392	392	65%	2.00E-110	29.93	QUE49099.1
[Botrytis cinerea RNA virus 1]	hypothetical protein	390	390	66%	9.00E-110	31.80	QOL11121.1
[Grapevine associated totivirus-2]	hypothetical protein	388	388	66%	7.00E-109	30.81	ADO60932.1
[Sclerotinia sclerotiorum dsRNA mycovirus-L-WX1]	hypothetical protein	384	384	63%	1.00E-107	31.15	QUE49144.1
[Sclerotinia sclerotiorum dsRNA mycovirus-L-WX2]	hypothetical protein	381	381	59%	2.00E-106	31.56	QUE49182.1
[Sclerotinia sclerotiorum dsRNA mycovirus-L]	hypothetical protein	377	377	65%	2.00E-105	30.40	YP_006331064.1
[Fusarium poae mycovirus 1]	hypothetical protein	375	375	61%	3.00E-104	30.41	YP_009272908.1
[Fusarium poae dsRNA virus 3]	hypothetical protein	374	374	65%	7.00E-104	29.12	YP_009253996.1

[Rosellinia necatrix fusagravirus 1]	hypothetical protein	372	372	69%	9.00E-103	29.37	BBB86777.1
[Phomopsis viticola mycovirus 961]	hypothetical protein	363	363	63%	3.00E-101	28.87	QWE35565.1
[Rosellinia necatrix fusagravirus 1]	hypothetical protein	365	365	59%	3.00E-100	30.26	AVD68672.2
[Sclerotinia sclerotiorum fusagravirus 2]	hypothetical protein	356	356	65%	5.00E-98	30.48	QUE49101.1
[Fusarium graminearum dsRNA mycovirus-3]	hypothetical protein	332	332	60%	2.00E-89	29.33	YP_003288788.1
[Monilinia virus D]	hypothetical protein	249	249	40%	2.00E-65	32.25	QED42929.1
[Caloscypha fulgens fusagravirus 1]	structural protein	191	191	50%	9.00E-45	26.06	QUP79365.1
[Monilinia virus E]	hypothetical protein	138	138	25%	2.00E-29	28.00	QED43011.1
[Sclerotium rolfsii RNA virus 1]	hypothetical protein	139	139	30%	4.00E-29	24.85	AZF86101.1
[Streptobotrys caulophylli fusagravirus 1]	hypothetical protein	135	135	44%	1.00E-27	24.43	UBQ34674.1
[Rosellinia necatrix mycovirus]	structural/gag protein	135	135	43%	1.00E-27	24.43	QMU26428.1
[Rosellinia necatrix fusagravirus 3]	putative structural protein	133	133	51%	6.00E-27	24.24	BBB86784.1
[Bremia lactucae associated fusagravirus 1]	ORF2	129	129	55%	1.00E-25	25.26	QIP68010.1
[Rosellinia necatrix fusagravirus 2]	putative structural protein	117	117	23%	4.00E-22	29.16	BBB86782.1
[Phlebiopsis gigantea mycovirus dsRNA 2]	hypothetical protein	106	106	28%	1.00E-18	24.28	CAJ34334.2
[Papaya meleira virus]	coat protein	100	100	48%	1.00E-16	20.37	AMU19319.1
[Picoa juniperi mycovirus 1]	hypothetical protein	72	72	28%	5.00E-08	20.71	QOI17265.1

Supplementary Table S3. BLASTP search with CcFGV1 ORF2-encoded RdRP.

Description (virus [isolate])	Description (protein)	Max score	Total score	Query cover	E value	Identity	Accession
[Cryphonectria naterciae fusagravirus 1]	RNA-dependent RNA polymerase	1071	1071	91%	0	45.83	UDM59941.1
[Fusarium virguliforme dsRNA mycovirus 1]	RNA-dependent RNA polymerase	767	767	96%	0	36.74	AEZ54148.1
[Fusarium virguliforme dsRNA mycovirus 2]	RNA-dependent RNA polymerase	760	760	98%	0	36.54	AEZ54146.1
[Trichoderma atroviride mycovirus]	RNA-dependent RNA polymerase	710	710	91%	0	35.55	YP_009342055.1
[Macrophomina phaseolina fusagravirus 1]	RNA-dependent RNA polymerase	587	587	93%	0	33.15	QDM35290.1
[Trichoderma asperellum dsRNA virus 1]	RNA-dependent RNA polymerase	560	560	95%	7.00E-173	31.12	YP_009553633.1
[Macrophomina phaseolina fusagravirus 3]	RNA-dependent RNA polymerase	553	553	95%	3.00E-170	31.16	QKO02086.1
[Macrophomina phaseolina fusagravirus 2]	RNA-dependent RNA polymerase	548	548	94%	6.00E-169	31.66	QKO02084.1
[Sclerotinia sclerotiorum dsRNA mycovirus-L-WX2]	RNA-dependent RNA polymerase	541	541	91%	2.00E-165	32.62	QUE49183.1
[Macrophomina phaseolina fusagravirus 4]	RNA-dependent RNA polymerase	536	536	87%	6.00E-165	32.06	QKO02088.1
[Sclerotinia sclerotiorum dsRNA mycovirus-L]	RNA-dependent RNA polymerase	539	539	91%	1.00E-164	32.37	YP_006331065.1
[Sclerotinia sclerotiorum dsRNA mycovirus-L-WX1]	RNA-dependent RNA polymerase	534	534	91%	5.00E-163	31.83	QUE49145.1
[Macrophomina phaseolina double-stranded RNA virus 2]	RNA-dependent RNA polymerase	526	526	78%	1.00E-162	33.30	ALD89097.1
[Macrophomina phaseolina fusagravirus 5]	RNA-dependent RNA polymerase	525	525	78%	5.00E-162	33.05	QKO02090.1
[Sclerotinia sclerotiorum dsRNA mycovirus-L]	RNA-dependent RNA polymerase	526	526	91%	9.00E-160	32.18	CEZ26307.1
[Rosellinia necatrix fusagravirus 1]	RNA-dependent RNA polymerase	522	522	98%	1.00E-158	29.58	BBB86778.1
[Sclerotinia sclerotiorum fusagravirus 2]	RNA-dependent RNA polymerase	521	521	90%	4.00E-158	31.62	QUE49102.1
[Rosellinia necatrix fusagravirus 1]	replicase	519	519	98%	2.00E-157	29.68	AZG06258.1
[Botrytis cinerea RNA virus 1]	RNA-dependent RNA polymerase	516	516	99%	4.00E-156	31.42	YP_009115498.1
[Sclerotinia sclerotiorum dsRNA mycovirus-L]	RNA dependent RNA polymerase	509	509	91%	1.00E-153	31.16	CEZ26308.1

[Fusarium poae dsRNA virus 3]	RNA-dependent RNA polymerase	486	486	84%	5.00E-145	32.83	YP_009253997.1
[Fusarium graminearum dsRNA mycovirus-3]	putative RNA-dependent RNA polymerase	460	460	91%	8.00E-136	31.03	YP_003288789.1
[Fusarium poae mycovirus 1]	RNA-dependent RNA polymerase	461	461	84%	1.00E-135	31.17	YP_009272909.1
[Fusarium poae dsRNA virus 2]	RNA-dependent RNA polymerase	457	457	98%	3.00E-134	29.00	YP_009253995.1
[Rosellinia necatrix fusagravirus 3]	RNA-dependent RNA polymerase	327	327	65%	5.00E-89	30.96	BBB86785.2
[Rosellinia necatrix fusagravirus 2]	RNA-dependent RNA polymerase	322	322	46%	1.00E-87	34.57	BBB86783.1
[Phlebiopsis gigantea mycovirus dsRNA 2]	putative RNA-dependent RNA polymerase	311	311	45%	5.00E-84	34.02	CAJ34335.2
[Picoa juniperi mycovirus 2]	RNA-dependent RNA polymerase	291	291	47%	5.00E-78	33.44	QOI17268.1
[Diplodia scrobiculata RNA virus 1]	RNA-dependent RNA polymerase	263	263	46%	6.00E-68	31.26	YP_003359178.1
[Papaya meleira virus]	RNA-dependent RNA polymerase	247	247	44%	9.00E-63	30.87	YP_009179230.1
[Picoa juniperi mycovirus 1]	RNA-dependent RNA polymerase	235	235	53%	4.00E-60	30.08	QOI17266.1
[Phytophthora infestans RNA virus 3]	RNA dependent RNA polymerasee	236	236	45%	1.00E-59	30.51	YP_009551328.1
[Papaya meleira virus]	RNA-dependent RNA polymerasee	226	226	42%	6.00E-56	29.10	AMU19320.1
[Hubei toti-like virus 11]	hypothetical protein	94	94	26%	3.00E-15	25.14	APG76021.1
[Lotchka virus]	RNA-dependent RNA polymerase	94	94	36%	3.00E-15	24.69	QRW42119.1
[Wuhan insect virus 28]	hypothetical protein 2	91	91	36%	6.00E-14	25.50	YP_009342430.1
[Totiviridae sp.]	RNA-dependent RNA polymerase	86	86	27%	1.00E-12	26.88	UCD53716.1
[Lotchka virus]	RNA-dependent RNA polymerase	86	86	36%	1.00E-12	24.28	QRW42121.1
[Lotchka virus]	RNA-dependent RNA polymerasee	86	86	36%	2.00E-12	24.28	QRW42117.1
[Rhizoctonia fumigata mycovirus]	putative RNA-dependent RNA polymerase	81	81	36%	8.00E-11	26.24	AJE29745.1
[Persimmon latent virus]	RNA-dependent RNA polymerasee	72	72	28%	4.00E-08	24.94	YP_009025166.1
[Sclerotinia sclerotiorum botybirnavirus 2]	cap-pol fusion protein	66	66	21%	2.00E-06	27.00	AMT92139.1
[Sclerotinia sclerotiorum botybirnavirus 2-WX]	cap-pol fusion protein	65	65	26%	6.00E-06	25.66	QUE49191.1

[Plasmopara viticola lesion associated dsRNA virus 1]	RNA dependent RNA polymerase	64	64	27%	1.00E-05	24.40	QGY93970.1
[Rosellinia necatrix quadrivirus 1]	RNA dependent RNA polymerase	57	57	22%	9.00E-04	23.81	BAM93353.1
[Barrymore virus]	RNA-dependent RNA polymerase	57	57	26%	0.001	25.46	QED21514.1
[Rosellinia necatrix quadrivirus 1]	RNA dependent RNA polymerase	55	55	26%	0.005	22.37	YP_005097975.1
[Spissistilus festinus virus 1]	RNA-directed RNA polymerase	53	53	26%	0.021	25.67	YP_003800001.1
